# Trajectories of Eating Behaviour Changes during Adolescence

**DOI:** 10.3390/nu13041313

**Published:** 2021-04-16

**Authors:** Radhouene Doggui, Stéphanie Ward, Claire Johnson, Mathieu Bélanger

**Affiliations:** 1 Centre de Formation Médicale du Nouveau-Brunswick (Université de Sherbrooke), Pavillon J.-Raymond-Frenette, Université de Moncton, 18 Antonine-Maillet Ave, Moncton, NB E1A 3E9, Canada; mathieu.belanger@umoncton.ca; 2 École des Sciences des Aliments, de Nutrition et d’Études Familiales, Université de Moncton, Moncton, NB E1A 3E9, Canada; stephanie.ward@umoncton.ca; 3 École des Hautes Études Publiques, Université de Moncton, Moncton, NB E1A 3E9, Canada; claire.johnson@umoncton.ca; 4 Department of Family and Emergency Medicine, Université de Sherbrooke, Sherbrooke, QC J1K 2R1, Canada; 5 Vitalité Health Network, Moncton, NB E2A 1A9, Canada

**Keywords:** adolescence, fruits and vegetables, sugary beverages, breakfast skipping, fast food, trajectory

## Abstract

Adolescence represents a critical transition phase during which individuals acquire eating behaviours that can track into adulthood. This study aims to characterise trends in eating behaviours throughout adolescence by investigating the presence of sub-groups of individuals presenting distinct trajectories of vegetable and fruit, sugary beverage, breakfast and fast-food consumption. Data from 744 MATCH study Canadian participants followed from 11 to 18 Years old (2013–2019) were included in the analyses. Participants reported how often they ate breakfast and consumed vegetables and fruits, sugary beverages and fast foods. Trajectories of eating behaviours over seven years were identified using group-based multi-trajectory modelling. For girls, three different groups were identified, namely ‘stable food intake with a decline in daily breakfast consumption’ (39.9%), ‘moderate food intake and worsening in overall eating behaviours’ (38.0%) and ‘stable high food intake’ (22.1%). For boys, five different groups were identified, namely ‘low food intake with stable daily breakfast consumption’ (27.3%), ‘breakfast-skippers and increasing fast food intake’ (27.1%), ‘low food intake with a decline in daily breakfast consumption’ (23.9%), ‘high food intake with worsening of eating behaviours’ (13.3%) and ‘average food intake with consistently high breakfast consumption’ (8.4%). Eating behaviours evolve through various distinct trajectories and sub-group-specific strategies may be required to promote healthy eating behaviours among adolescents.

## 1. Introduction

The transition from late childhood to adolescence is marked by significant eating behaviour changes [1]. Population-based studies have found that some eating behaviours, such as vegetable and fruit consumption, skipping breakfast [2], consuming sugary beverages [3] and eating fast foods [4], worsen as children enter adolescence. For example, a multi-national study conducted in the Eastern Mediterranean region showed a negative trend between age and the prevalence of eating vegetables and fruits ≥5 times per day among adolescents [5]. Further, a national Canadian study found that while 98% of children 4–8 years old consumed breakfast daily this percentage decreased to 91% among 9–13-year-olds and 82% among 14–18-year-olds [2]. Conversely, fast food consumption tends to increase during that same period. A longitudinal study in the United States found that fast food consumption increased from 2 times per week to 3 times per week between the ages of 11 and 18 [4]. Similarly, national data in the United States have shown that the prevalence of heavy sugary beverages consumption increased with age, where 5% of children as compared to 16% of adolescents consumed more than 500 calories from these beverages daily [3].

Findings from previous cross-sectional and longitudinal studies suggest that these eating behaviours are inter-dependant and may co-develop [6]. For example, studies have linked irregular breakfast consumption to lower intakes of vegetables and fruits among adolescents, and have shown that frequent fast food consumption is strongly correlated with skipping breakfast among this age group [7,8,9]. Fast food consumption has also been shown to be associated with a greater intake of sugary beverages [10]. However, for some behaviours, the relationship remains unclear. While one cross-sectional study among 15,283 Texan middle and high school students found that sugary beverages were associated with increased consumption of unhealthy foods (e.g., unhealthy meats, fried snacks, desserts), this study also found that soda intake was associated with lower consumption of vegetables and fruits, while flavoured and sports drinks were associated with a greater vegetable and fruit intake [11].

The emergence of group-based latent trajectory methods can be particularly interesting for advances in nutritional epidemiology research as it accounts for ‘between-individual’ variation when used to describe the continuity of different behaviours in a cohort of individuals over time. This addresses a limitation of previous longitudinal analyses, which described trends in dietary behaviours as a group average without acknowledging that sub-groups of individuals may present development patterns that vary from the group average. In particular, population-based trajectory models do not consider clusters of individuals who follow similar eating behaviour trajectories and who could be at a greater health risk than others [12]. Few studies have considered between-individual variation and the potential that different groups of individuals follow distinct developmental trajectories for different behaviours [6,13]. For example, the recent use of group-based latent class analyses has helped identify important differences in how sub-groups of individuals follow distinct trends regarding adherence to the Mediterranean diet [14], traditional or modern diets in China. Further, a study by Chen and Hsiao [6] identified two heterogeneous eating behaviour trajectories among girls and two among boys between the ages of 9 and 13. Specifically, this Taiwanese study found that one group of girls was characterised by consistent daily consumption of breakfast with a shift from high to low vegetable and fruit intake while a second group presented irregular breakfast consumption and low vegetable and fruit intake. Among boys, one group was characterised by having a consistent daily consumption of breakfast and a high intake of vegetables and fruits, while the second group presented irregular breakfast consumption and a low intake of vegetables and fruits. These studies highlight the importance of considering the potential for various trajectories when describing the development of eating behaviours. Moreover, these studies call for a better understanding of the co-development of different eating behaviours among boys and girls during adolescence. Information on multi-behaviour co-development could help identify intervention requirements specific to adolescent sub-groups.

This study aimed to investigate the presence of naturally occurring sub-groups of girls and boys following distinct trajectories of multiple eating behaviours (i.e., daily breakfast consumption, fast food consumption, vegetable and fruit intake, and sugary beverage intake). We hypothesised that several different patterns of development of eating behaviours would be identified; at least a group with a worsening of the majority of eating behaviours and another group with a healthy profile for the development of eating behaviours. Finally, we expected to find that an improvement in one eating behaviour would correlate with the improvements in other eating behaviours.

## 2. Methods

### 2.1. Study Population

This study used data collected from 937 children (mean age 10.8 years old, in Grades 5 and 6 at study inception) recruited from 17 elementary schools in New Brunswick, Canada for the Monitoring Activities for Teenagers to Comprehend their Habits (MATCH) longitudinal study [15]. Detailed information on the MATCH study protocol has been published elsewhere [15], but briefly, students completed a self-reported questionnaire three times per academic year, over 8 years from 2011 to 2019. They came from schools selected to represent geographical (i.e., rural and urban), and cultural (i.e., French and English) diversity. Questions related to students’ eating behaviours were included once per academic year, starting in the second year of data collection. Therefore, seven cycles (cycles 5, 8, 11, 14, 17, 20 and 23) of data were available for analysis.

### 2.2. Eating Behaviours

For this study, eating behaviours included daily breakfast consumption, fast food consumption, vegetable and fruit intake, and sugary beverage intake. For breakfast and fast food consumption, participants were asked to identify the number of days (0 to 7) over the course of the previous week they had eaten breakfast and had eaten a meal or snack at a fast food restaurant. Participants were then categorised as daily breakfast eaters (i.e., eating breakfast seven times during the previous week) or not [16]. Vegetable and fruit intake was assessed by asking ‘How many times in the past 7 days have you eaten…’, followed by 5 food items (i.e., fruit, salad, potatoes, carrots, other vegetables). Similarly, participants were asked ‘How many times in the past 7 days have you drank…’, followed by 9 beverage items (i.e., fruit juice, soft drinks, diet soft drinks, sports drinks, energy drinks, coffee/tea or other coffee-flavoured products, sugar-sweetened beverages, water and milk). For these items, response options included: ‘I did not eat (drink) this in the past 7 days’, ‘1 to 3 times in the past 7 days’, ‘4 to 6 times in the past 7 days’, ‘1 time per day’, ‘2 times per day’, ‘3 times per day’ and ‘4 times per day or more’ [17,18]. The questionnaire showed good test-retest reliability (kappa ≥ 0.61) over two weeks [19]. All responses were converted to daily frequency equivalents. For response options with a range, the middle value of the interval was used in analyses such that ‘1 to 3 times in the past 7 days’ was represented as 0.29 times per day, ‘4 to 6 times in the past 7 days’ was transformed to 0.71 times per day, and ‘4 times per day or more’ was expressed as 4 times per day. Vegetable and fruit intake was determined by calculating the sum of the frequency of intake of all items in this category. Sugary beverage intake represented the sum of all beverages with the exclusion of coffee and tea, water and milk.

### 2.3. Data Analysis

#### 2.3.1. Descriptive Analysis

For both genders, median frequency change over time for vegetable and fruit intake, daily breakfast consumption and fast food consumption was assessed by fixed effect quantile regression [20]. Fixed-effect Poisson regression [21] was used to evaluate the frequency of sugary beverage intake over time. The age of participants was used as the time variable and was introduced as a categorical independent variable. The assessment of linear and quadratic trends was done by orthogonal polynomial contrasts.

#### 2.3.2. Group Based Multi-Trajectory Modelling

The group-based multi-trajectory group modelling approach developed by Nagin et al. [22] was used to assess the co-development of the eating behaviours (*Traj* command in Stata 16.1 (Stata Corporation, College Station, TX, USA, 2019), [23,24]). This approach is based on the application of finite-mixture models which assume that a population is composed of a mixture of distinct groups that could be differentiated by their distinct trajectories of behaviour development [12]. Participants who had complete data on at least two measurement points were retained for this analysis. Given that the Shapiro-Wilk test pointed to a non-normal distribution of the ‘vegetable and fruit intake ’, ‘sugary beverage intake’ and ‘fast food consumption’ variables, these variables were modelled by a beta distribution as recommended by Elmer et al. [25]. The ‘daily breakfast consumption’ variable was expressed as the probability of adherence to the recommendation of having breakfast every day (7 days a week) and modelled as a logit distribution. Group-based multi-trajectory group modelling was conducted separately for girls and boys [26]. The optimal number of groups was based on the best-fitting model using the Bayesian Information Criterion. Then, various polynomial functions were tested for each latent trajectory class to determine if they should be modelled as linear, quadratic or cubic patterns of change throughout adolescence. Before retaining a model, we verified that the requirements of minimum mean posterior probability of group was ≥70%, odds of correct classification ≥5 and that each group included ≥5% of participants in the sample. Following the identification of the trajectory groups, a label was assigned to each group to characterise their pattern of overall eating behaviours. Finally, in the interest of parsimony, the models presented do not include adjustments for culture (francophone or anglophone) or geographical location (rural or urban) since preliminary analyses indicated these variables provided no additional meaningful information.

## 3. Results

### 3.1. Description of the Study Sample

Of the 937 participants recruited in the MATCH study, 744 provided data during at least two of the survey cycles when questions on eating behaviours were administered and were therefore retained for these analyses. Of these participants, 44.2% were boys, 52.4% attended a school in a rural area, and two-thirds attended a Francophone school.

### 3.2. Average Eating Behaviour Trends

Among girls, the median (Table 1) frequency of vegetable and fruit intake, sugary beverage intake and daily breakfast consumption were characterised by a marked linear decline throughout adolescence. Boys also reported a linear decline in daily breakfast consumption over time whereas their sugary beverage intake increased in early adolescence before dropping to lower levels by the end of follow-up.

### 3.3. Eating Behaviour Trajectories

**Figure 1:**
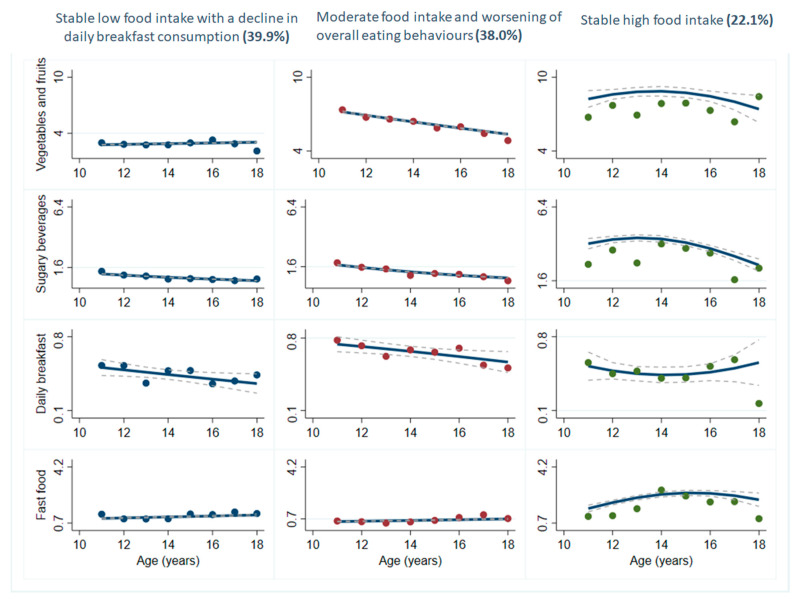
Multi-trajectory modelling of four eating behaviours among adolescent girls in the MATCH study. Figure caption: MATCH: Monitoring Activities of Teenagers to Comprehend their Habits. A dashed line represents the 95% confidence interval. Solid lines represent the average frequency of daily intakes for vegetables and fruits, sugary beverages and fast food, and the probability of daily breakfast consumption.

Three groups were identified to present distinct trajectories of multiple eating behaviours among girls, whereas five groups emerged among boys (Table 2). No group emerged as presenting consistent healthy eating behaviours among girls or boys. The largest group among girls included 40% of participants and was labelled ‘*stable low food intake with a decline in daily breakfast consumption’* as it was characterised by a very low intake of vegetables and fruits, sugary beverages and fast food (Figure 1). Adherence to the recommendation to consume breakfast daily declined consistently within this group. The second group contained nearly as many participants (38%) and was labelled ‘*moderate food intake and worsening of overall eating behaviours*’ given it showed a significant decrease in vegetable and fruit intake, sugary beverage intake and daily breakfast consumption. The 3rd group displayed the lowest proportion (22.1%) of girls and was labelled ‘*stable high food intake*’. Girls belonging to this group showed stable and relatively high frequencies for all eating behaviours.

**Figure 2:**
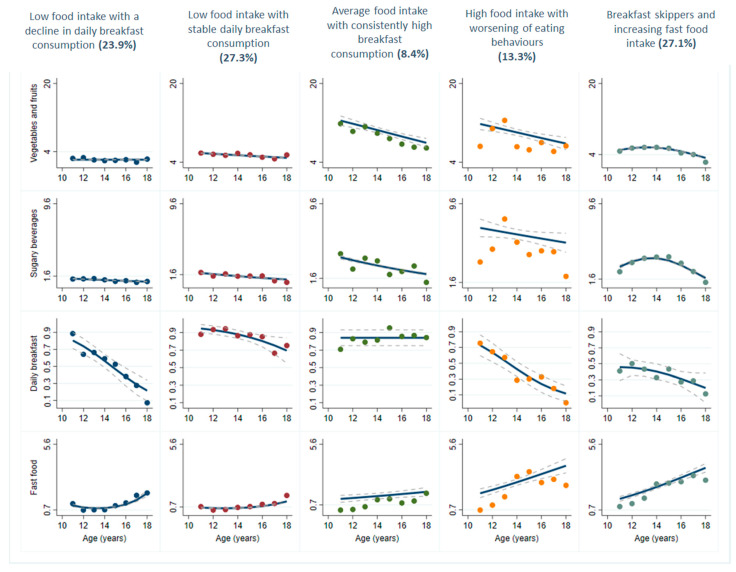
Multi-trajectory modelling of four eating behaviours among adolescent boys in the MATCH study. Figure caption: MATCH: Monitoring Activities of Teenagers to Comprehend their Habits. Dashed line represents the 95% confidence interval. Solid lines represent the average frequency of daily intakes for vegetables and fruits, sugary beverages and fast food, and the probability of daily breakfast consumption.

Among boys, the largest group included 27.3% of participants and was labelled ‘*Low food intake with stable daily breakfast consumption*’. Participants in this group had a low vegetable and fruit intake and low fast food consumption throughout follow-up (Figure 2). This group was also characterised by a low and declining intake of sugary beverages and a high, but declining adherence to daily breakfast consumption. A second group also represented 27.1% of participants and was labelled ‘*breakfast-skippers and increasing fast food intake*’. It included boys with the lowest probability of eating breakfast daily. Boys belonging to this group also presented a substantial increase in fast food consumption and a consistently low intake of vegetables and fruits. A group including 24% of boys was labelled ‘*low food intake with a decline in daily breakfast consumption*’ showed stable and low intakes of vegetables, fruits and sugary beverages. This group also had a sharp decline in daily breakfast consumption. The ‘*high food intake with worsening of eating behaviours’* group included 13% of participants and showed a relatively high intake of sugary beverages, an increase in fast food consumption and a decline in vegetable and fruit intake. The smallest group of boys (8% of participants) was labelled ‘*average food intake with consistently high breakfast consumption*’. This group showed high and consistent daily breakfast consumption but was otherwise similar to other groups with regards to the other eating behaviours.

## 4. Discussion

### 4.1. Key Findings

This study is the first to use multi-trajectory groups to illustrate how four common eating behaviours evolve throughout adolescence. Our study found a total of eight different eating behaviour group trajectories; three among girls and five among boys. None of the groups demonstrated consistent healthy eating behaviours. Among both genders, average trends showed a worsening of most eating behaviours, with declines in daily breakfast consumption emerging in almost every group. Our findings also showed that while some eating behaviours may improve in some adolescents, this does not necessarily translate to improvements in other eating behaviours. These findings not only demonstrate the need for nutrition interventions in adolescence, but also the diversity and complexity of eating behaviour development among this age group.

### 4.2. Breakfast and Fast Food Trajectories

Of the four eating behaviours assessed, daily breakfast consumption appears to be the most unstable as it declined in six of the eight trajectory groups identified. Other Canadian studies also found low adherence to daily breakfast recommendations among adolescents. In one study, 48.5% of Canadian adolescents were found to skip breakfast at least once a week [27] and another study reported a general decline in breakfast consumption with age during adolescence [2]. Adolescents may therefore be missing out on the many nutritional and health benefits of regular breakfast consumption [4,28,29]. In particular, skipping breakfast has been linked to increased feelings of hunger [30], which may lead to greater consumption of high-energy foods [31]. This may partly explain why our study found that for three of the sub-groups among boys (64.3%), a decline in breakfast consumption coincided with an increase in fast food consumption. In addition to being of low nutritional quality, fast foods are typically considered ultra-processed foods, which have been linked to overconsumption, increased caloric intake and ultimately, weight gain [32]. The common co-development of these two behaviours (skipping breakfast and fast food consumption) raises concern not only because of their effect on weight [4] but also because of their potential compounding effect on short and long-term health outcomes (e.g., obesity) [33]. It may therefore be strategic to develop interventions targeting both regular breakfast consumption and low fast food intake simultaneously among adolescent boys.

### 4.3. Vegetables and Fruits Trajectory

In general, girls reported higher intakes of vegetables and fruits than boys. Nevertheless, four out of eight groups displayed consistently low intakes of vegetables and fruits and three other groups showed a steep decline in vegetable and fruit consumption during adolescence. Our findings are consistent with previous cross-sectional studies that showed that Canadian adolescents generally consume insufficient amounts of vegetables and fruits [34] and that this behaviour seems to worsen as adolescents become older [35]. Only one group among girls met the recommended intake for vegetables and fruits, which aligns with findings from previous studies [34,36]. For example, an international cross-sectional study showed that fruit consumption was more common among Canadian girls compared to boys in early and mid-adolescence [36]. However, this contrasts with a 2004 to 2015 trend analysis reporting a higher frequency of vegetable and fruit intake among boys (2.96 to 2.40 servings per day) than girls (2.5 to 1.93 servings per day) [37]. Considering the importance of vegetables and fruits for adolescents’ optimal growth, development and overall health, our findings highlight the importance of continuing to promote the consumption of these foods to the majority of adolescents.

### 4.4. Sugary Beverages Trajectory

In contrast with other eating behaviours, we noted an improvement in the majority of groups (six of eight) concerning the consumption of sugary beverages. The decline in sugary beverages observed in our sample may reflect a trend observed throughout Canada, where sugary beverage intake was found to drop from 2004 to 2015 [38,39]. Although trends characterizing sugary beverage intake are improving, these beverages remain a concerning source of sugar and total energy intake in Canadian children and adolescents [39]. Our results also align with the recent national data showing that sugars represent nearly one-quarter of Canadian adolescents’ daily energy intake [39], which is more than double the 5–10% recommended by the World Health Organization [40]. Considering the short and long-term health consequences of high sugar intakes [41], public health messaging should therefore continue to encourage adolescents to decrease their intake of sugars, particularly from beverages.

### 4.5. Public Health Significance

While this study demonstrates changes in eating behaviours during adolescence, it is difficult to establish a specific time point or age when these changes occur. However, changes in eating behaviours often correspond with the transition from middle school to high school, where adolescents have more autonomy regarding their food choices and eating behaviours [42]. Adolescence is also typically a time when concerns regarding body image emerge, which can lead to unhealthy eating behaviours such as reducing food intake and skipping meals in an effort to lose weight [43]. In fact, one Canadian study found that 58% of adolescent girls reported ‘trying to lose weight’ as their weight goal [43]. This could help explain why declines in sugary beverages, daily breakfast, as well as vegetables and fruits, were observed in a greater proportion of girls than boys sub-groups in our study. Considerations such as these, and gender-specific findings in this study, highlight the need for gender-specific approaches during adolescence as has been suggested previously [44].

Further, the identification of different groups among boys and girls, and the observation that each group presents at least one unhealthy eating behaviour trajectory, underscores the complexity of addressing adolescents’ eating behaviours from a population perspective. Contrary to expectations, improvements in one eating behaviour did not necessarily correlate with improvements in other eating behaviours. As such, it is unlikely that simple public health messages (i.e., consuming more vegetables and fruits or eating breakfast daily) would be sufficient to improve adolescents’ overall diet. Considering the heterogeneity in eating behaviours of adolescents, it may be necessary to address various eating behaviours simultaneously. Coupling general public health messages with more targeted interventions may be required to address the co-development of multiple eating behaviours during adolescence. Public health authorities are urged to intervene as early as possible, namely during childhood or early adolescence to prevent the worsening of eating behaviours. Additionally, there is evidence that gender-specific interventions may be needed to improve adolescents’ nutrition. Since gender differences in eating behaviour trajectories might be explained by differences in girls’ and boys’ perception of what constitutes a healthy diet [45], public health initiatives should focus on improving nutrition knowledge while also addressing adolescents’ perceived barriers to healthy eating.

Previous studies suggested that eating behaviours track from adolescence to early adulthood [46]. While this study did not assess behaviours in adulthood, results suggest that during adolescence, eating behaviours can track in some individuals but not all. Future analyses should investigate which adolescents’ eating behaviours track in adulthood. Future studies should also seek to identify determinants of the various eating behaviour trajectory classes. Additional information on characteristics associated with the various groups will help inform public health interventions.

### 4.6. Strengths and Limitations

The present study had several strengths, including the relatively large sample and the inclusion of multiple measures over seven years spanning the transition from late childhood through adolescence. In addition, this study moved away from presenting general trends for the entire sample. Further, it accounted for the co-development of four common eating behaviours, which provides a greater understanding of the complexity of eating behaviour development throughout adolescence.

Our study also has some limitations. First, since participants reported how many times they consumed a food rather than the number of servings, is not possible to know if answers provided corresponded with standard serving sizes, nor did it provide information on daily caloric intake. Second, the use of a self-report questionnaire may have resulted in under- or over-reporting due to recall and social desirability biases [47]. Third, while this study did consider four important eating behaviours of adolescents, it did not consider all of them (e.g., snacking/grazing, distractive eating, dieting, etc.,). Finally, the sampling design of this study does not allow for generalisations among all adolescents.

## 5. Conclusions

This study allowed identifying multiple patterns of eating behaviour development throughout adolescence. These patterns are generally characterised by a worsening of eating behaviours during adolescence, except for sugary beverage intake, which stabilised or decreased over the seven years of the study. Further highlighting the complexity of promoting healthy eating behaviours in adolescence, this study also showed that eating behaviour trajectories differed between girls and boys, and therefore calls for the need to develop gender-specific public health interventions that focus on multiple eating behaviours. Further research is needed to identify factors that influence the co-development of eating behaviour trajectories.

## Figures and Tables

**Table 1:** Median frequencies of eating behaviours from childhood to adolescence.

Eating Behaviours	11 Years (*n* = 309)		12 Years (*n* = 587)		13 Years (*n* = 579)		14 Years (*n* = 497)		15 Years (*n* = 445)		16 Years (*n* = 419)		17 Years (*n* = 347)		18 Years (*n* = 47)		*P* Trend	
	Median	IQR ^1^	Median	IQR ^1^	Median	IQR ^1^	Median	IQR ^1^	Median	IQR ^1^	Median	IQR ^1^	Median	IQR ^1^	Median	IQR ^1^	Linear	Quadratic
	Overall (*n* = 744)																	
Vegetable and fruit (daily)	4.0	2.3–6.7	4.4	2.3–7.3	4.3	2.0–7.0	4.0	2.0–6.6	4.1	2.0–6.7	3.9	2.0–6.4	3.6	1.6–5.7	4.0	1.7–5.6	0.058	0.68
Sugary beverage (daily)	2.0	1.0–4.3	1.7	0.9–4.0	1.9	0.9–4.0	1.3	0.6–4.0	1.3	0.3–3.6	1.3	0.3–3.3	1.09	0.3–2.1	0.6	0.0–2.0	<0.0001	<0.0001
Breakfast (Daily)	7.0	5.0–7.0	7.0	5.0–7.0	7.0	4.0–7.0	7.0	4.0–7.0	7.0	4.0–7.0	7.0	4.0–7.0	6.0	4.0–7.0	6.0	3.0–7.0	<0.0001	0.56
Fast food (Daily)	1.0	0.0–1.0	1.0	0.0–1.0	1.0	0.0–1.0	1.0	0.0–2.0	1.0	0.0–2.0	1.0	0.0–2.0	1.0	0.0–2.0	1.0	0.0–2.0	<0.0001	0.96
	Girls (*n* = 415)																	
Vegetable and fruit (daily)	4.7	2.3–6.7	4.3	2.3–7.0	4.1	2.1–6.5	4.1	2.1–6.3	4.1	2.7–6.6	4.0	2.3–6.4	3.9	1.7–5.7	4.0	1.9–6.0	<0.0001	0.49
Sugary beverage (daily)	1.7	1.0–4.0	1.3	0.7–3.6	1.3	0.7–3.0	1.0	0.3–2.1	1.0	0.3–2.6	1.0	0.3–1.9	0.7	0.3–1.3	0.4	0.0–1.0	<0.0001	0.034
Breakfast (Daily)	7.0	5–7	7.0	4.0–7.0	6.0	3.0–7.0	7.0	4.0–7.0	7.0	4.0–7.0	7.0	4.0–7.0	6.0	4.0–7.0	5.5	3.0–7.0	0.042	0.39
Fast food (Daily)	1.0	0.0–1.0	1.0	0.0–1.0	0.0	0.0–1.0	1.0	0.0–2.0	1.0	0.0–2.0	1.0	0.0–2.0	1.0	0.0–2.0	1.0	0.0–1.0	0.12	0.72
	Boys (*n* = 329)																	
Vegetable and fruit (daily)	3.9	2.1–6.7	4.7	2.1–7.7	4.4	1.9–7.3	4.0	2.0–7.0	4.0	1.6–7.0	3.3	1.4–6.6	3.0	1.4–5.4	3.0	1.3–6.0	0.41	0.83
Sugary beverage (daily)	2.1	1.0–4.9	2.3	1.0–4.7	3.0	1.0–6.0	2.5	1.0–5.4	2.1	0.7–4.3	2.1	1.0–4.9	1.6	0.3–3.3	1.0	0.3–2.4	<0.0001	<0.0001
Breakfast (Daily)	7.0	6.0–7.0	7.0	6.0–7.0	7.0	5.0–7.0	7.0	4.0–7.0	7.0	4.0–7.0	7.0	4.0–7.0	6.0	4.0–7.0	5.5	2.5–7.0	<0.0001	0.47
Fast food (Daily)	1.0	0–1.0	0.0	0.0–1.0	1.0	0.0–1.0	1.0	0.0–2.0	1.0	0.0–3.0	1.0	0.0–3.0	2.0	0.0–3.0	1.5	0.0–3.0	0.39	0.94

^1^ Interquartile range.

**Table 2:** The estimated group-specific trajectory regression coefficients for the group-based multi-trajectory model (*n* = 744).

Group	%	Term	Vegetables and Fruits		Sugary Beverages		Breakfast		Fast Food	
			Estimate ± s.e. ^1^	*p* Value	Estimate ± s.e.	*p* Value	Estimate ± s.e.	*p* Value	Estimate ± s.e.	*p* Value
		Girls (*n* = 415)								
1	39.9	Intercept	−2.00 ± 0.20	<0.0001	−1.50 ± 0.25	<0.0001	1.01 ± 0.61	0.063	−2.16 ± 0.32	<0.0001
		Linear	0.02 ± 0.01	0.27	−0.10 ± 0.02	<0.0001	−0.09 ± 0.04	0.026	0.03 ± 0.02	0.15
2	38.0	Intercept	0.09 ± 0.24	0.71	−0.53 ± 0.27	0.050	2.26 ± 0.68	0.0009	−3.02 ± 0.31	<0.0001
		Linear	−0.06 ± 0.02	0.0002	−0.14 ± 0.02	<0.0001	−0.11 ± 0.05	0.017	0.05 ± 0.02	0.036
3	22.1	Intercept	−3.42 ± 3.62	0.34	−5.70 ± 3.29	0.083	6.36 ± 5.98	0.36	−9.18 ± 3.67	0.012
		Linear	0.46 ± 0.52	0.37	0.72 ± 0.47	0.12	−0.93 ± 0.85	0.35	1.13 ± 0.53	0.032
		Quadratic	−0.02 ± 0.02	0.36	−0.02 ± 0.02	0.099	0.03 ± 0.03	0.35	−0.04 ± 0.02	0.047
Model goodness of fit										
BIC (*N* = 7192)			5116.7							
BIC (*N* = 415)			5172.3							
AIC			5250.8							
Log-likelihood			5289.8							
		Boys (*n* = 329)								
1	23.9	Intercept	−2.12 ± 0.09	<0.0001	−1.91 ± 0.41	<0.0001	5.66 ± 1.19	<0.0001	6.48 ± 3.78	0.086
		Linear	–	–	−0.04 ± 0.03	0.11	−0.38 ± 0.08	<0.0001	−1.27 ± 0.54	0.018
		Quadratic	–	–	–	–	–	–	0.04 ± 0.02	0.012
2	27.3	Intercept	−0.49 ± 0.28	0.078	−1.15 ± 0.37	0.0021	6.10 ± 1.54	0.0001	2.32 ± 3.25	0.47
		Linear	−0.03 ± 0.02	0.072	−0.08 ± 0.027	0.0016	−0.29 ± 0.10	0.0037	−0.73 ± 0.46	0.11
		Quadratic	–	–	–	–	–	–	0.03 ± 0.02	0.082
3	8.4	Intercept	1.94 ± 0.87	0.025	0.06 ± 0.64	0.92	1.66 ± 0.34	<0.0001	−2.37 ± 0.83	0.0043
		Linear	−0.59 ± 0.05	0.025	−0.11 ± 0.04	0.013	–	–	0.07 ± 0.05	0.19
		Quadratic	–	–	–	–	–	–	–	–
4	13.3	Intercept	1.62 ± 0.08	0.051	0.04 ± 0.77	0.61	5.71 ± 1.41	0.0001	−2.91 ± 0.79	0.0003
		Linear	−0.11 ± 0.06	0.046	−0.06 ± 0.05	0.30	−0.43 ± 0.10	<0.0001	0.17 ± 0.05	0.0018
		Quadratic	–	–	–	–	–	–	–	–
5	27.1	Intercept	−6.36 ± 3.22	0.048	−10.56 ± 3.25	0.0012	−3.21 ± 7.89	0.68	−3.57 ± 0.60	<0.0001
		Linear	0.82 ± 0.46	0.077	1.38 ± 0.46	0.0030	0.55 ± 1.13	0.62	0.20 ± 0.04	<0.0001
		Quadratic	−0.03 ± 0.01	0.060	−0.050 ± 0.016	0.0020	−0.02 ± 0.04	0.53	–	–
Model goodness of fit										
BIC ^2^ (*N* = 5301)			2921.1							
BIC (*N* = 329)			3007.9							
AIC ^3^			3125.5							
Log-likelihood			3187.5							

^1^ Standard error. ^2^ Bayesian information criterion. ^3^ Akaike information criterion.

## Data Availability

The datasets generated during and/or analyzed during the current study are not publicly available to ensure confidentiality and that any secondary analyses correspond to the objectives of the research project but are available from the corresponding author on reasonable request.
